# *eIF6* deficiency regulates gut microbiota, decreases systemic inflammation, and alleviates atherosclerosis

**DOI:** 10.1128/msystems.00595-24

**Published:** 2024-09-03

**Authors:** Zhenzhen Wang, Shuai Yang, Linglin Tong, Xin Li, Weiyi Mao, Honghua Yuan, Yang Chen, Shenyang Zhang, He Zhang, Renjin Chen

**Affiliations:** 1 Cancer Institute, Xuzhou Medical University, Xuzhou, China; 2 College of Life Sciences, Xuzhou Medical University, Xuzhou, Jiangsu, China; 3 School of Basic Medical Sciences, Nanjing Medical University, Jiangsu, China; 4 Department of Neurology, the Affiliated Hospital of Xuzhou Medical University, Xuzhou, Jiangsu, China; Southern Medical University, Guangzhou, Guandong, China

**Keywords:** *eIF6 *gene, atherosclerosis, gut microbiota, metabolites, *L. acidophilus*

## Abstract

Altered composition of the gut microbiota affects immunity and metabolism. This study previously found that *eIF6* gene knockdown changes the composition of the intestinal flora in the *eIF6* gene knockdown mouse model. *Lactobacillus acidophilus* is significantly increased in the model. This study was designed to investigate the role of *L. acidophilus* in the pathogenesis of atherosclerosis. Transcriptomic data from 117 patients with coronary artery disease (CAD) and 79 healthy individuals were obtained. *ApoE*^−/−^ and *ApoE*^−/−^/*eIF6*^+/−^ mice on normal chow diet or a high-fat diet were treated for 16 weeks; *eIF6* deficiency was evaluated atherosclerosis. *ApoE*^−/−^ mice on normal chow diet or a high-fat diet were treated with *L. acidophilus* by daily oral gavage for 16 weeks. Moreover, one group was treated with lipopolysaccharide at 12 weeks. The levels of *eIF6*, *RNASE3*, and *RSAD2* were notably higher in the patients with CAD than in the healthy individuals. *eIF6* deficiency altered the composition of gut microbiota. *eIF6* deficiency reduced the atherosclerotic lesion formation in *ApoE*^−/−^/*eIF6*^+/−^ mice compared with the *ApoE*^−/−^ mice. The microbial sequencing and metabolomics analysis demonstrated some beneficial bacterial (*L. acidophilus*, *Ileibacterium*, and *Bifidobacterium*) and metabolic levels significantly had deference in *ApoE*^−/−^/*eIF6*^+/−^ mice compared with the *ApoE*^−/−^ mice. Correlational studies indicated that *L. acidophilus* had close correlations with low-density lipoprotein cholesterol, lesion area, and necrotic area. *L. acidophilus* inhibited high-fat diet-induced inflammation and atherosclerotic lesion, increasing the expression of tight junction proteins (ZO-1 and claudin-1) and reducing the gut permeability. However, lipopolysaccharide reversed the protective effect of *L. acidophilus* against atherosclerosis. *eIF6* deficiency protected against atherosclerosis by regulating the composition of gut microbiota and metabolites. *L. acidophilus* attenuated atherosclerotic lesions by reducing inflammation and increasing gut permeability.

**IMPORTANCE:**

*eIF6* deficiency modulates the gut microbiota and multiple metabolites in atherosclerotic *ApoE*^*−/−*^ mice. *L. acidophilus* was reduced in the gut of atherosclerotic *ApoE*^*−/−*^ mice, but administration of *Lactobacillus acidophilus* reversed intestinal barrier dysfunction and vascular inflammation. Our findings suggest that targeting individual species is a beneficial therapeutic strategy to prevent inflammation and atherosclerosis.

## INTRODUCTION

Atherosclerosis, the leading cause of cardiovascular diseases, is a chronic inflammatory disease that develops within the walls of arterial vessels, characterized by lipid deposition, inflammatory cell infiltration, plaque rupture, and thrombus formation as its main features (1, 2). Cytokines of the innate and adaptive immune systems play important roles in all stages of atherogenesis (3). The gut microbiota plays a crucial role in regulating the functioning of the innate and adaptive immune systems (4). Modifying the composition of the gut microbiota has been linked to systemic inflammation, ultimately contributing to the development of metabolic diseases such as obesity, diabetes, and atherosclerosis (5, 6). The presence of lipopolysaccharide (LPS) derived from the microbiota resulted in the progression of atherosclerosis, but the absence of microbiota attenuated atherosclerosis development (7). Studies have provided evidence of the presence of bacterial DNA within atherosclerotic plaques, suggesting a potential involvement of the gut microbiota in the progression of atherosclerosis (8). The germ-free apolipoprotein E (*ApoE*^−/−^) mice showed accelerated atherosclerotic lesions with a high-fat diet (HFD) compared with the conventionally raised mice (9). Inhibition of trimethylamine-N-oxide (TMAO) as produced by the gut microbiota has been shown to reduce the risk of developing atherosclerosis (10). It indicates that gut microbiota is closely related to atherosclerosis.

Eukaryotic initiation factor 6 (*eIF6*) has been identified as a translation factor that acts as a rate-limiting step for achieving optimal protein synthesis in response to insulin or growth factors (11). *In vitro* studies have demonstrated that depleting *eIF6* leads to a decrease in lipid accumulation and an increase in fatty acid oxidation. Similarly, *eIF6* knockout mice exhibited reduced white fat deposits (12, 13). We found that *eIF6* regulated the fatty acid synthase to inhibit the atherosclerosis (14). *eIF6* deficiency decreases the systemic inflammation by shaping gut microbiota, which remains unknown.

In the study, we explore the relationship of *eIF6* and gut microbiota with atherosclerosis. *eIF6* deficiency promotes production of beneficial bacteria, the abundance of *Lactobacillus acidophilus* in *ApoE*^−*/*−^/*eIF6^+/^*^−^ mice compared with *ApoE*^−^*^/^*^−^ mice with HFD. Therefore, we further investigated the protective effect of *L. acidophilus*.

## MATERIALS AND METHODS

### RNA sequencing data processing

The RNA sequencing data used in this study were obtained from the Gene Expression Omnibus (https://www.ncbi.nlm.nih.gov/geo/). The following keywords were used: “coronary artery disease” [MeSH]), “gene profiling,” and “blood.” Consequently, GSE221911 and GSE190627 were selected for subsequent analyses (15, 16).

### Transcriptome analysis

Patients with coronary artery disease (CAD; *n* = 117) and healthy controls (*n* = 79) were analyzed. Briefly, raw reads (FASTQ files) were checked for quality using FastQC software and filtered to remove low-quality calls using Trimmomatic (17). Processed reads were then aligned to the human reference genome (GRCh38) using STAR software. The HTSeq count algorithm with default parameters (gene annotation release 109 from Ensembl) was used to produce gene counts for each sample (18). Differential expression analysis was performed using the DESeq2 package (19). Read counts were normalized by calculating the size factor, as implemented in DESeq2. Therefore, 15,787 genes were tested for differential expression. Differentially expressed genes (DEGs) were selected by considering a *P* value of <0.05 and |log_2_(fold change [FC])| >0.35. Analyses were performed using R v.4.2.1.

### Animal model

*ApoE*^−/−^/*eIF6^+^*^/−^ double transgenic mice were generated by crossing *ApoE*^−/−^ and *eIF6^+^*^/−^ mice. *eIF6^+^*^/−^ mice on a C57BL/6J background were obtained from San Raffaele Scientific Institute (Milan, Italy). *ApoE*^−/−^ mice on a C57BL/6J background were purchased from GemPharmatech Co. Ltd., Nanjing, China. Eight-week-old *ApoE*^−/−^ and *ApoE*^−/−^/*eIF6^+^*^/−^ mice were fed either a normal chow diet or high-fat diet for 16 weeks. Osmotic pumps with lipopolysaccharide (200 µg/kg body weight per day) or phosphate-buffered saline (PBS) were subcutaneously implanted into *ApoE*^−/−^ mice at 12 weeks.

### Culture and administration of *Lactobacillus acidophilus* CICC 6075

*L. acidophilus* CICC 6075 (China Industrial Microbial Strain Collection Management Center) was isolated from the gastrointestinal tract and cultured in De Man, Rogosa, and Sharpe media for a period of 48 h at 37°C under anaerobic conditions. The absorbance at the wavelength of 600 nm was used to measure the concentration of bacteria. *L. acidophilus* (5 × 10^9^ CFU) in 100 µL of PBS was orally gavaged daily to *ApoE*^−/−^ mice.

### Quantitative analysis of atherosclerotic lesions

The whole aortas were dissected longitudinally along the small curvature, and the dissected aorta was placed in 4% paraformaldehyde for overnight fixation. Then the aortas were washed three times with pure water, then moistened with 60% isopropyl alcohol for 2 min. Oil Red O working solution (saturated Oil Red O solution dissolved in isopropyl alcohol mixed with distilled water 3:2 and filtered) and stained for 1 h. Aortic root was embedded in OCT to prepare sections (6 µm), washed with 60% isopropanol for 2 min, stained with Oil Red O working solution for 10 min, toned with 60% isopropanol, then rinsed with ice water, left in hematoxylin solution for 5 min, and rinsed again with running water, and the stained sections were embedded in glycerol gelatin. The aortic mass and root were photographed and recorded using a microscope system (Leica). Lesion size was measured on the microscope photographs using ImageJ software.

### DNA extraction and 16S rRNA gene sequencing

Fecal samples were taken, promptly frozen in liquid nitrogen, and then kept at −80°C for storage. Using the Fast DNA Stool Mini Kit (Qiagen, Hilden, Germany), we isolated bacterial genomic DNA from stool samples. The 16S rRNA gene’s hypervariable V3–V4 region was extracted by PCR using the primer sequences 338F and 806R, which stand for 5′-ACTCCTACGGGAGGCAGCAG-3′ and 5′-GGACTACHVGGGTWTCTAAT-3′, respectively. The amplified DNA was then sequenced using Illumina’s MiSeq platform (San Diego, CA, USA). The software program Quantitative Insights into Microbial Ecology (QIIME2 v.2021.11) was used to process the 16S rRNA sequencing data. The sequencing reads were filtered and a feature table was created using the DADA2 algorithm included within QIIME2. The Silva (SSU138) 16S rRNA database (v.13.8) was used to perform taxonomic categorization and cluster amplicon sequence variants (ASVs) at 97% sequence identity. The Kruskal–Wallis test was utilized to compare different alpha-diversity indexes. Unweighted UniFrac distance measurements were used in beta-diversity analysis to examine variations in microbial community structure between samples. For visualization, principal coordinate analysis (PCoA) was employed. With a minimal linear discriminant analysis (LDA) score of 4, the linear discriminant analysis effect size (LEfSe) was used to find biomarkers for both numerous taxa and functional pathways. The R package v.4.2.1 was used to create heatmaps. PICRUSt, based on ASVs, was used to estimate the prevalence of functional categories using Kyoto Encyclopedia of Genes and Genomes orthologs (KOs) (20).

### Intestinal permeability assessment

Mice were given DX-4000 FITC (Sigma-Aldrich, St. Louis, MO, USA) orally in the dose of 600 mg/kg after a 12-h fast. Four hours later, orbital blood collection was used to get serum from the animals. After incubating the serum for 30 min, it was centrifuged for 20 min at 800 × *g*. PBS was used to dilute the resultant serum by 10-fold. Using a fluorescence spectrophotometer (Synergy H1, BioTek, Winooski, VT, USA) with an excitation of 485 nm and an emission of 535 nm, the amount of DX-4000 FITC in the serum was determined.

### Western blotting

The mice intestinal tissue was homogenized and then lysed in RIPA buffer (Meilunbio, MA0151) supplemented with protease inhibitors (Meilunbio, MB2678) to prepare protein lysates. The proteins were separated using SDS-PAGE, transferred onto a polyvinylidene fluoride membrane (Millipore Corporation, IPVH00010), and blocked using 5% milk without fat for 1 h at room temperature. After blocking, the membrane was incubated with claudin-1 (Proteintech, 13050-1-AP, dilution rate: 1:1,000) or ZO-1 (Proteintech, 21773-1-AP, dilution rate: 1:5,000) and actin (Proteintech, 23660-1-AP, dilution rate: 1:5,000) antibody in 5% milk overnight at 4°C. Secondary antibodies, including anti-rabbit immunoglobulin G (IgG) (Bioworld, BS13278, dilution rate: 1:5,000) or anti-mouse IgG (Bioworld, BS12478, dilution rate: 1:5,000), were diluted 5,000 times. For immunodetection, an enhanced chemiluminescence Western blotting substrate (Beyotime, P0018S) was used, and the signals were detected using a Tanon-4600 imaging system.

### Hematoxylin–eosin staining

The embedded paraffin mice intestinal tissue was cut at 6 µm for hematoxylin–eosin staining. The tissue sections underwent deparaffinization and rehydration and were subsequently subjected to a 7-min hematoxylin staining. The sections were rinsed using running tap water and stained with eosin for 30 seconds. After dehydration, the sections were mounted and captured.

### Immunofluorescent staining

To perform immunofluorescence staining on paraffin-embedded sections of mice, intestinal tissue underwent deparaffinization, rehydration, and heat-mediated antigen retrieval using 1 mM EDTA (Beyotime, C0196), blocked using 5% bovine serum albumin (Solarbio, A8020) for 1 h at room temperature. Secondary antibodies, including claudin-1 (Proteintech, 13050-1-AP, dilution rate: 1:100) or ZO-1 (Proteintech, 21773-1-AP, dilution rate: 1:200) overnight at 4°C. Subsequently, the sections were incubated with a horseradish peroxidase-conjugated secondary antibody targeting rabbit IgG (Cell Signaling Technology) and visualized using DAB (ZSGB-BIO, ZLI9017). The positively stained area was manually outlined and quantified using ImageJ software.

### Statistical analysis

All data are expressed as mean ± SEM. Data are analyzed using Student’s *t*-test or one-way analysis of variance followed by Tukey’s multiple comparison as a post hoc test. A *P* value of <0.05 was considered significant. All data analyses were performed using GraphPad Prism v.8.0.

## RESULTS

### Data preprocessing and identification of DEGs

**Fig 1:**
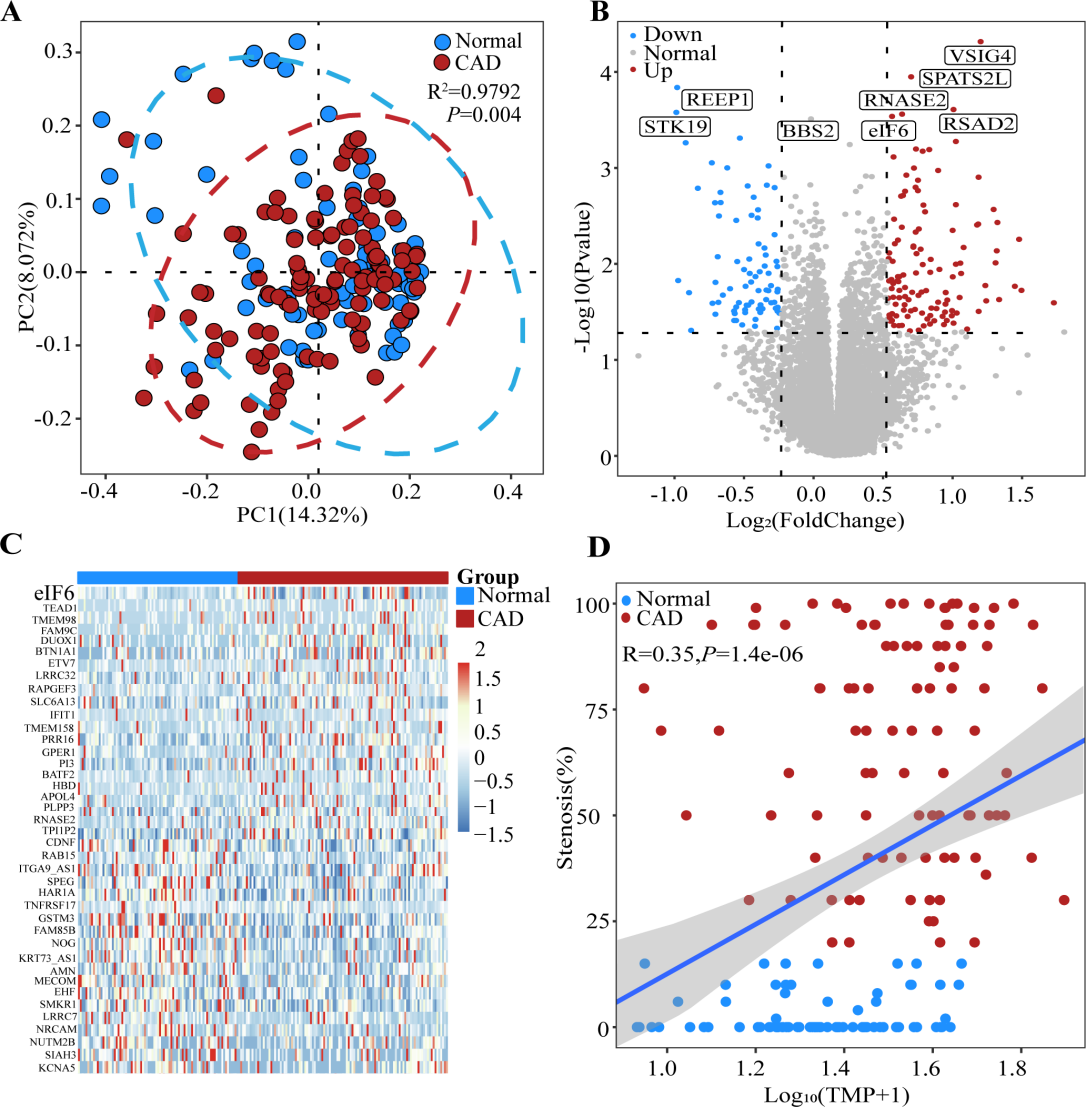
*eIF6* levels are decreased in the blood of patients with CAD. (**A**) Principal coordinate analysis of RNAseq of whole blood from the healthy control and CAD groups using Bray–Curtis (*R*^2^ = 0.9792, *P* = 0.004) distance. (**B**) Volcano plot displaying the statistical significance (*P* value) versus magnitude of change (fold change) of protein-coding genes and transcripts in the CAD group compared with the healthy control group. DEGs/isoforms with |log_2_(FC)| > 0.35 are labeled. (**C**) Cluster heatmap plot of the top 40 DEGs in each sample. (**D**) Pearson’s correlation between stenosis (%) and normalized expression of *eIF6*. Shadow represents the 95% confidence interval. Data are expressed as means ± SEM. Normal = 79, CAD = 117. CAD, coronary artery disease; DEG, differentially expressed gene.

After merging the data, 117 patients with CAD and 79 healthy controls were obtained. With a threshold of |log_2_(FC)| of >0.35 and an adjusted *P* value of <0.05, we identified 202 DEGs (122 upregulated and 80 downregulated). PCoA results demonstrated significant differences between the CAD and control samples (Fig. 1A). Specifically, the expression levels of *eIF6*, *RNASE3*, *RSAD2*, *BPATS3L*, and *BSIG4* were significantly higher in the patients with CAD than in the healthy controls (Fig. 1B). Additionally, the cluster heatmap plot and Pearson’s correlation suggested that *eIF6* was highly expressed in the patients with CAD (Fig. 1C and D).

### *eIF6* deficiency alleviated atherosclerotic progression

**Fig 2:**
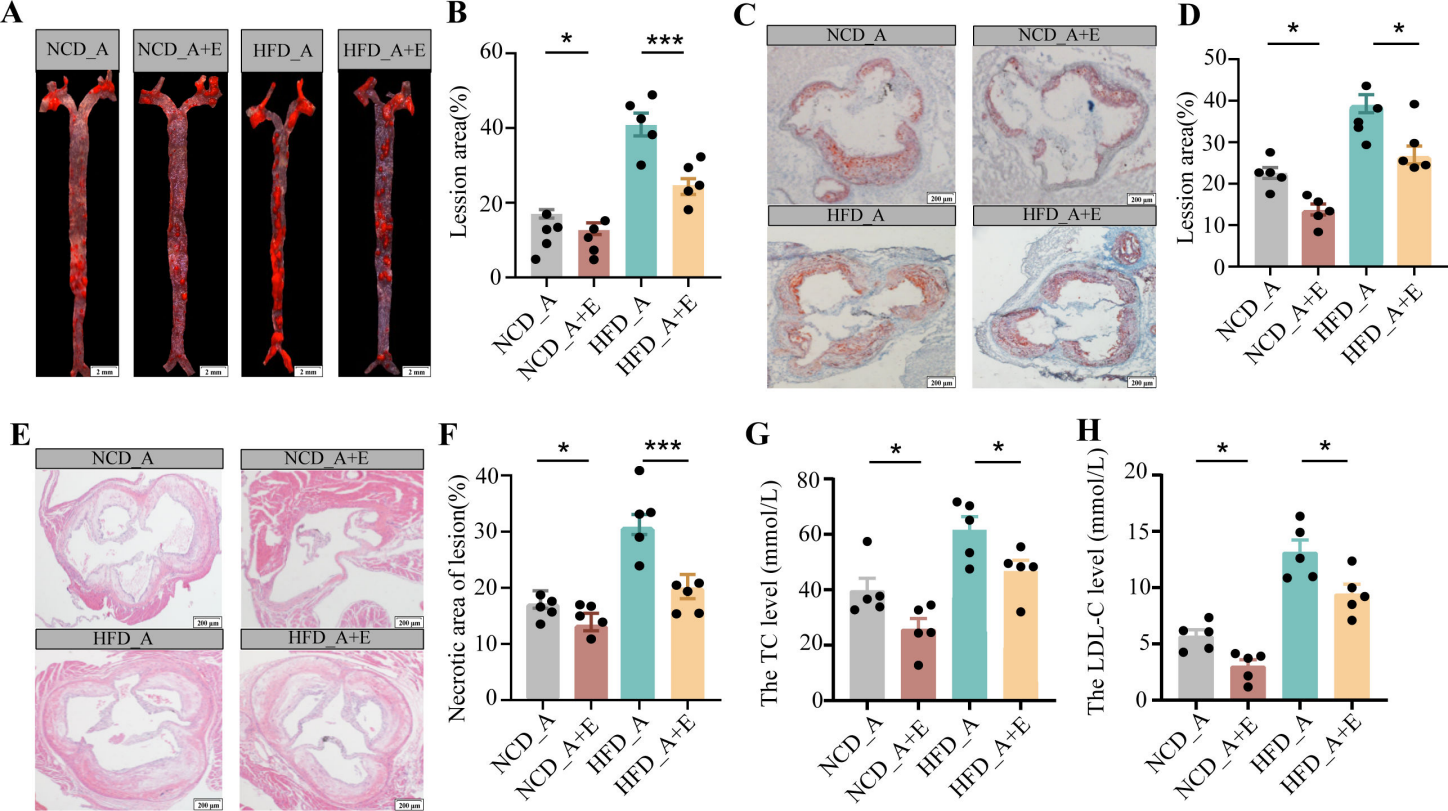
*eIF6* deficiency reduced atherosclerotic progression. (A and B) *ApoE*^−/−^ and *ApoE*^−/−^/*eIF6*^+/−^ mice were fed NCD and HFD for 16 weeks, and atherosclerosis in the aorta was visualized by Oil Red O staining. *n* = 6, scale bar = 1 mm. (C and D) Aortic root sections from *ApoE^−/−^* and *ApoE^−/−^*/*eIF6*^+/−^ mice fed NCD and HFD for 16 weeks were stained with Oil Red O. *n* = 6, scale bar = 100 µm. (E and F) Aortic root sections from *ApoE^−/−^* and *ApoE^−/−^*/*eIF6*^+/−^ mice fed NCD and HFD for 16 weeks were stained with hematoxylin and eosin. *n* = 6, scale bar = 100 µm. Serum triglyceride level (**G**), Serum LDL-C level (**H**). Representative images of each group are shown. Data (B–G) are shown as the mean ± SEM (*P < 0.05, **P < 0.01, ***P < 0.001) by one-way analysis of variance and Tukey’s post hoc analysis (NCD_A, NCD_*ApoE*^−/−^, NCD_A + E, NCD_*ApoE*^−/−^/*eIF6*^+/−^, HFD_A, HFD_*ApoE*^−/−^, HFD_A + E, HFD_*ApoE*^−/−^/*eIF6*^+/−^). LDL-C, low-density lipoprotein cholesterol; NCD, normal chow diet; TC, total cholesterol.

We want to validate whether *eIF6* deficiency can alleviate atherosclerosis. *ApoE^−^*^/^^−^/*eIF6*^+/−^ mouse model was successfully established. The experiment animals were grouped as follows: normal chow diet (NCD)*-ApoE^−^*^/−^, NCD-*ApoE*^−/−^/*eIF6^+^*^/−^, HFD-*ApoE^−^*^/−^, and HFD-*ApoE^−^*^/−^/*eIF6^+^*^/−^ groups. The atherosclerotic lesion in HFD-*ApoE*^−/^*^−^*/*eIF6^+^*^/−^ mice was significantly decreased compared to HFD-*ApoE*^−/−^ mice in aortas and aortic root regions (Fig. 2A through D). *eIF6* deficiency decreased the necrotic core area in atherosclerotic plaques (Fig. 2E and F) and reduced the content of serum triglyceride (TG) and low-density lipoprotein cholesterol (LDL-C) (Fig. 2G and H). Although there was no significant difference in the content of serum total cholesterol (TC) and high-density lipoprotein cholesterol (HDL-C). According to these data, we found that *eIF6* deficiency alleviates atherosclerosis and improves a stable plaque phenotype.

### *eIF6* deficiency altered the gut microbiota composition

**Fig 3:**
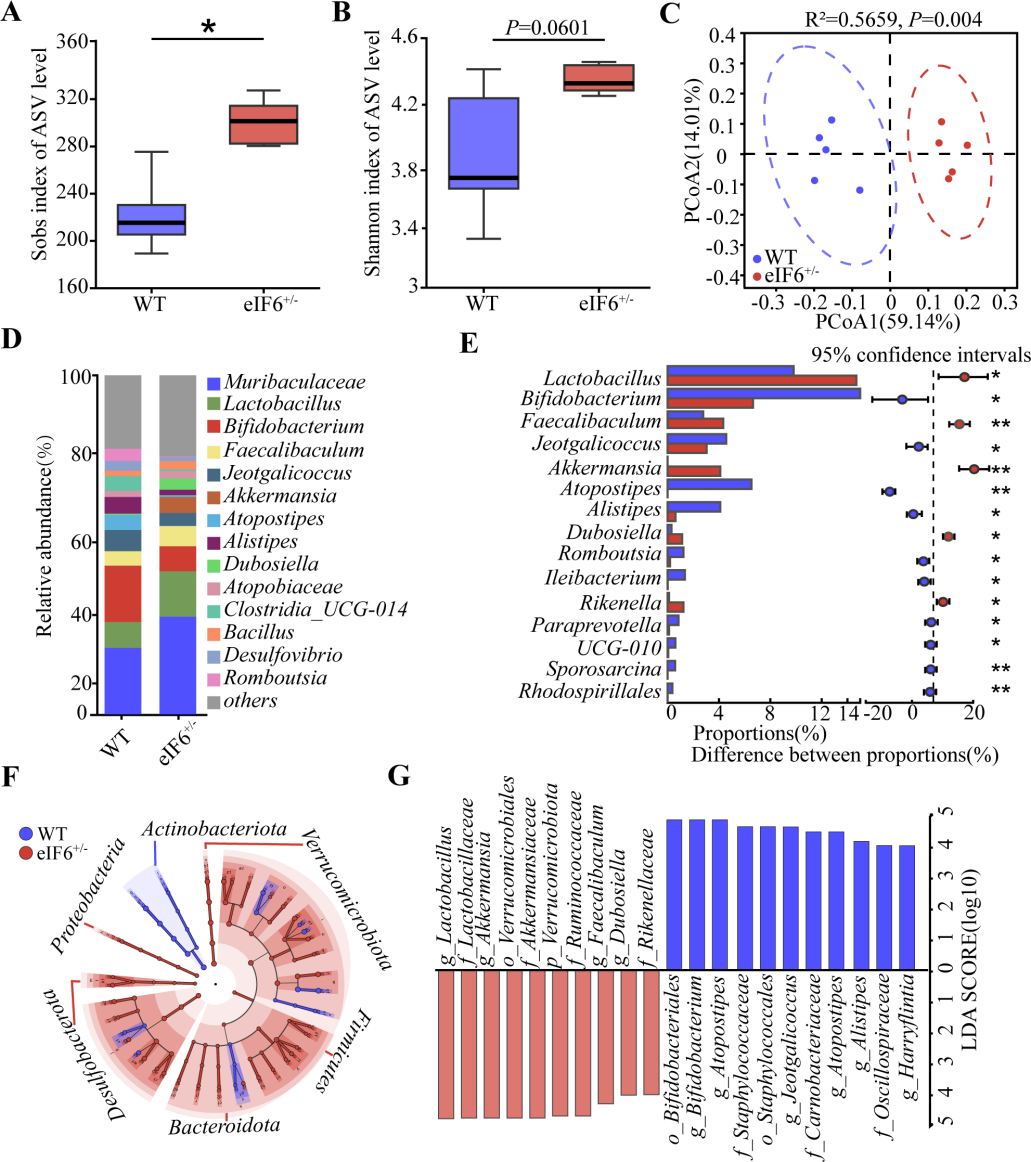
*eIF6* deficiency changed fecal microbial composition in mice. α-Diversity of the gut microbiome from two groups: (**A**) Sobs index and (**B**) Shannon index. (**C**) PCoA of all samples by weighted UniFrac distance. (**D**) The percentage of community abundance on the genus level in different groups. (**E**) Systems-theoretic accident model and processes (STAMP) analysis of bacterial genus differences between the two groups. (**F**) Cladogram generated from linear discriminant analysis effect size showing the most diferentially enriched bacterial taxa in the fecal microbiota of C57 (blue) or *eIF6*^+/−^ (red) mice (LDA value = 4, *P* < 0.05). (**G**) Computed LDA scores of the relative abundance difference between the C57 and *eIF6^+/−^* groups. Negative LDA scores (red) are enriched in the C57 group, while positive LDA scores (blue) are enriched in the *eIF6*^+/−^ group. Data are shown as the mean ± SEM (*P < 0.05) by one-way analysis of variance and Tukey’s post hoc analysis. LDA, linear discriminant analysis; PCoA, principal coordinate analysis; WT, wild type.

To explore the effect of *eIF6* deficiency on the intestinal microbiota, 16S rDNA gene sequencing was performed on the feces of C57BL/6J and *eIF6^+/^*^−^ mice. Higher bacterial species richness was observed in the *eIF6^+/^*^−^ mice compared with the control mice (Fig. 3A). The diversity index (Shannon) in the *eIF6*^+/−^ mice tended to be higher than that in the control mice (*P* = 0.0601, Fig. 3B). Based on the results of principal component analysis (Fig. 3C), a clear clustering pattern emerged in the microbiota composition of both C57BL6/J and *eIF6*^+/−^ mice. The stacked bar chart further demonstrates the prevalence of specific genera within the composition. *Muribaculaceae*, *Lactobacillus*, and *Bifidobacterium* were enriched in both C57BL/6J and *eIF6*^+/−^ mice. The relative abundance of *Lactobacillus* was significantly increased in the *eIF6^+/^*^−^ mice, while the abundance of *Bifidobacterium* was significantly decreased in the *eIF6*^+/−^ mice (Fig. 3D and E). To identify the bacterial taxa affected by *eIF6*, we employed the LEfSe method (Fig. 3F). The analysis revealed significant variations in the composition of intestinal bacterial communities between the different groups. Furthermore, using LDA with a threshold of LDA > 4, we were able to identify the major taxa that exhibited significant differences between C57BL/6J and *eIF6^+/^*^−^ mice.The top three groups eliminated by *eIF6* were *o_Bifidobacteriales*, *g_Atoposipes*, and *f_Staphylococcaceae*, whereas those enriched by *eIF6* were *Lactobacillus*, *g_Akkermansia* and o_Verrucomicrobiales (Fig. 3G).

### *eIF6* deficiency altered the composition of gut microbiota in *ApoE*^−/−^ mice

**Fig 4:**
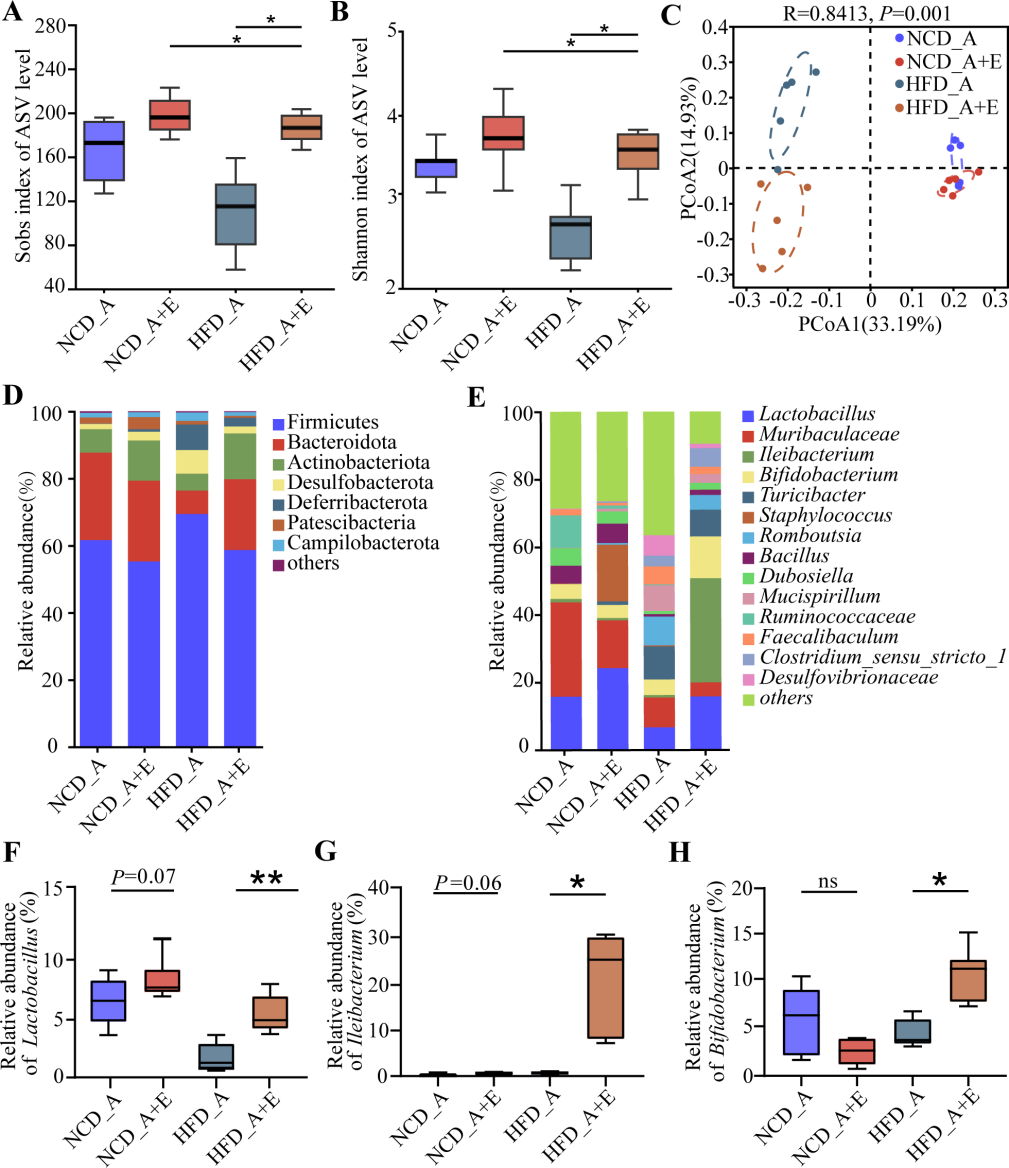
*eIF6* deficiency changed fecal microbial composition in *ApoE*^−/−^ mice. α-Diversity of the gut microbiome from four groups. (**A**) Sobs index. (**B**) Shannon index. (**C**) PCoA of all samples by weighted UniFrac distance. (**D**) The percentage of community abundance on phylum level in NCD_A, NCD_A + E, HFD_A, or HFD_A + E groups. (**E**) The percentage of community abundance on the genus level in NCD_A, NCD _A + E, HFD_A, or HFD_A + E groups. (**F**) Relative abundance of *Lactobacillus* in feces of mice in NCD_A, NCD_A + E, HFD_A, or HFD _A + E groups. (**G**) Relative abundance of *Ileibacterium* in feces of mice in NCD_A, NCD_A + E, HFD_A, or HFD_A + E groups. (**H**) Relative abundance of *Bifidobacterium* in feces of mice in NCD_A, NCD_A + E, HFD_A, or HFD_A + E groups. Data (A, B, and G–H) are shown as the mean ± SEM (*P < 0.05, **P < 0.01, ***P < 0.001) by one-way analysis of variance and Tukey’s post hoc analysis. ns, not significant; PCoA, principal coordinate analysis.

In order to investigate the potential role of the gut microbiota in mediating the beneficial effects of *eIF6* on alleviating atherosclerosis development, we performed 16S rDNA gene sequencing on fecal samples of mice that were administered a standard NCD or HFD treatment. Lower bacterial species richness and diversity were observed in HFD_A + E-treated mice than in HFD_A mice but not in NCD_A + E compared with NCD_A (Fig. 4A and B).

Principal component analysis showed distinct clustering of the microbiota composition in mice treated with NCD_A, NCD_A + E, HFD_A, and HFD_A + E (Fig. 4C). In total, Firmicutes and Bacteroidetes were the dominant phylum in the NCD_A, NCD_A + E, and HFD_A groups. However, Firmicutes and Actinobacteria were the dominant phyla in the HFD_A + E group (Fig. 4D). According to the stacked bar chart, the genus with the highest relative abundance was consistently dominant among the NCD_A, NCD_A + E, and HFD_A groups (relative abundance > 5%, Fig. 4E). The relative abundance of *Lactobacillus*, *Ileibacterium*, and *Bifidobacterium* was significantly higher in the HFD_A + E mice compared with in the HFD_A mice, while no significant difference was found between the NCD_A and NCD_A + E mice (Fig. 4F and H).

To distinguish the particular types of bacteria present in various conditions and to compare the microbiota composition among distinct groups, we employed linear discriminant analysis effect size. The cladogram demonstrated the presence of 22 taxa within the four experimental groups (Fig. S2A), revealing substantial variations in the compositions of intestinal bacterial communities across different groups. Furthermore, the LDA (>4) effectively identified the major taxa exhibiting significant differences among the four groups. The results indicated that the relative abundance of *Bacteroidota*, *Muribaculaceae*, and *Oscillospirales* was higher in the NCD_A group; the relative abundance of *Coriobacteriales*, *Atopobiaceae*, and *Patescibacteria* was higher in the NCD_A + E group; the relative abundance of *Clostridia*, *Turicibacter*, and *Peptostreptococcale* was higher in the HFD_A group; and the relative abundance of *Erysipelotrichaceae*, *Bacilli*, and *Ileibacterium* was higher in the HFD_A + E group (Fig. S2B).

### *eIF6* deficiency altered the serum metabolites in *ApoE*^−/−^ mice

**Fig 5:**
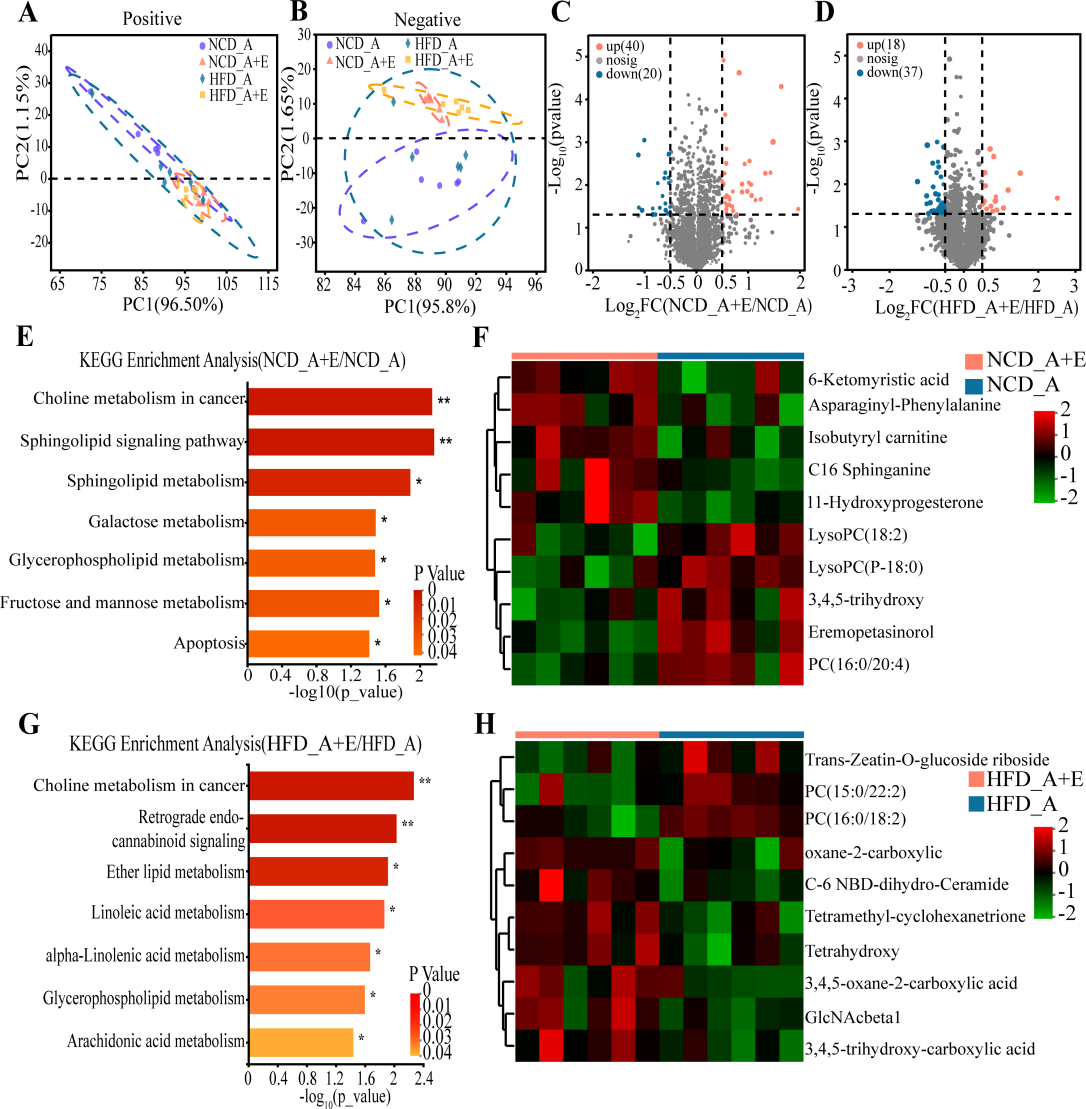
*eIF6*^+/−^ changed serum metabolites in *ApoE*^−/−^ mice. Analysis of serum metabolites in different groups based on PCoA analysis (unweighted UniFrac distance). (**A**) Positive ion mode. (**B**) Negative ion mode. (**C**) Volcano plot showing differential serum metabolites of mice between the NCD_A and NCD_A + E groups. (**D**) Volcano plot showing differential serum metabolites of mice between the HFD_A and HFD _A + E groups. (**E**) The KEGG pathway analysis result of differential serum metabolites of mice between the NCD_A_A and NCD_A _A + E groups. (**F**) Top 10 differential serum metabolites of mice between the NCD_A and NCD_A + E groups. (**G**) The KEGG pathway analysis result of differential serum metabolites of mice between the HFD _A and HFD _A + E groups. (**H**) Top 10 differential serum metabolites of mice between the HFD _A and HFD _A + E groups.

Using untargeted metabolomics profiling on fasting serum samples, we observed that *eIF6* knockdown led to significant metabolic alterations compared with the control group, regardless of standard NCD or HFD (Fig. 5A and B). We successfully identified a total of 60 differential metabolites that meet the criteria of variable importance in projection (VIP) of >1.0 and *P* < 0.05, highlighting the notable differences between the groups NCD_A + D and NCD_A (Fig. 5C). The differential metabolites between the NCD_A + D and NCD_A groups underwent a pathway enrichment analysis using the Kyoto Encyclopedia of Genes and Genomes (KEGG), there was significant enrichment observed in several pathways, such as choline metabolism in cancer, sphingolipid signaling pathway, galactose metabolism, glycerophospholipid metabolism, fructose and mannose metabolism, and apoptosis (Fig. 5E). There were 55 differential metabolites screened out between HFD_A + D and HFD_A (Fig. 5D). The KEGG pathways results showed that choline metabolism in cancer, retrograde endocannabinoid signaling, ether lipid metabolism, linoleic acid metabolism, alpha-linolenic acid metabolism, glycerophospholipid metabolism, and arachidonic acid metabolism were enriched between HFD_A + D and HFD_A (Fig. 5G).

**Fig 6:**
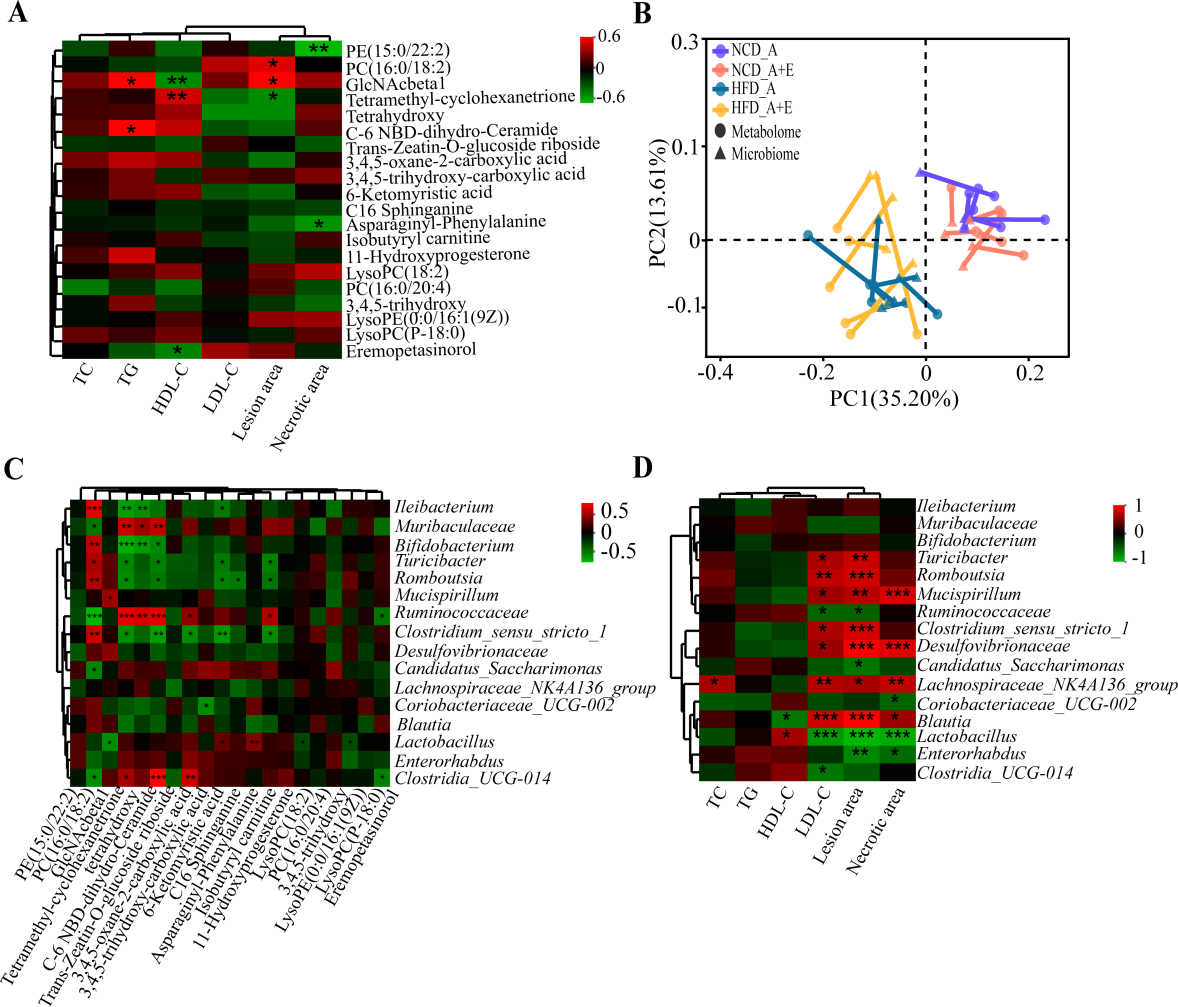
Correlation analysis between differential microbiota and serum metabolites. (**A**) Association between the serum differential metabolites and clinical indices. (**B**) Procrustes analysis showed that the correlation between microbiota and serum metabolites. (**C**) Associations between the serum differential microbiota and microbiota. (**D**) Association between the microbiota and clinical indices. Data are shown as the mean ± SEM. *P < 0.05, **P < 0.01, ***P < 0.001.

Correlation analysis of different serum metabolites, lesion area, and necrotic area was conducted to further determine the effects of serum metabolites on atherosclerosis. Results showed that the GlaNAcbetal was significantly positively correlated with TG and lesion area and significantly negatively correlated with HDL-C (Fig. 6A). A significant correlation was observed between the differential microbiota and the differential metabolites by Procrustes analysis (Fig. 6B). From this, the correlations between the dominant genus (relative abundance >5%) and different serum metabolites were analyzed, and results showed that GlaNAcbetal was significantly positively correlated with *Mucispirillum* and significantly negatively correlated with *Lactobacillus* (Fig. 6C). Additional analysis revealed significant positive correlations between *Lactobacillus* and HDL-C levels, as well as significant negative correlations with LDL-C levels, lesion area, and necrotic area (Fig. 6D). Overall, *Lactobacillus* could contribute to alleviating the development of atherosclerosis.

### *L. acidophilus* protected against atherosclerotic lesion formation in *ApoE^−/−^* mice

**Fig 7:**
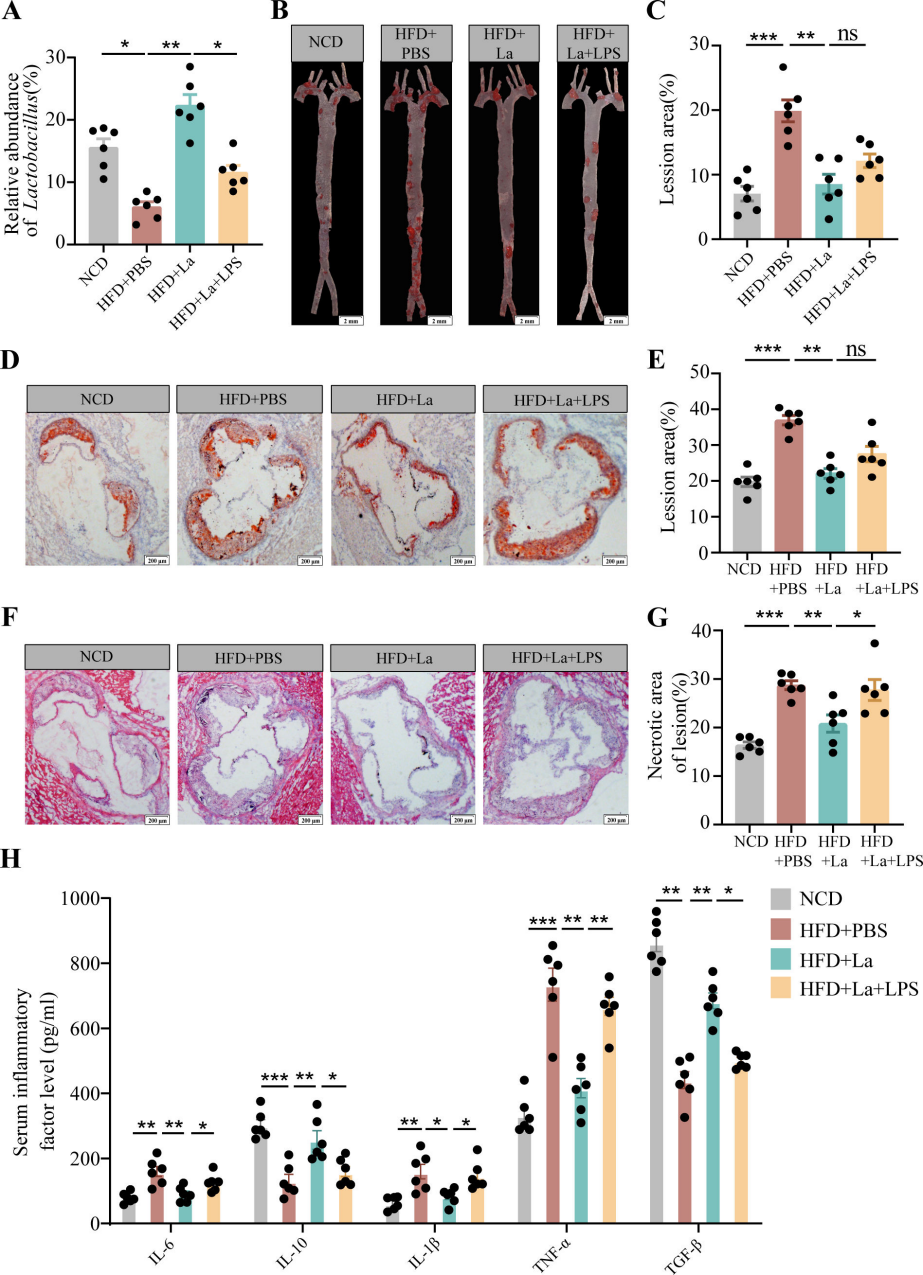
*L. acidophilus* improves atherosclerotic progression. (**A**) The relative abundance of *L. acidophilus* was measured by real-time PCR in the stool of four groups (NCD, HFD + PBS, HFD + La, and HFD + La + LPS) mice (*n* = 6). (**B and C**) The four groups (NCD, HFD + PBS, HFD + La, and HFD + La + LPS) were fed either an NCD or Western diet for 16 weeks. The lipid content of the aorta was visualized by staining with Oil Red O. *n* = 6, scale bar = 2 mm. (**D and E**) Aortic root sections were analyzed by Oil Red O. *n* = 6, scale bar = 100 µm. (**F and G**) Aortic root sections were analyzed by hematoxylin and eosin staining. *n* = 6, scale bar = 100 µm. (**H**) Inflammatory factors were detected by enzyme-linked immunosorbent assay. Representative images of each group are shown. (D–H) Data are shown as the mean ± SEM (*P < 0.05, **P < 0.01, ***P < 0.01) by one-way analysis of variance and Tukey’s post hoc analysis. IL, interleukin; NCD, normal chow diet; TGF-β, transforming growth factor beta; TNF-α, tumor necrosis factor alpha.

*ApoE*^−/−^ mice (four groups) were fed normal chow diet or high-fat diet with live *L. acidophilus* or PBS by oral gavage daily for a period of 16 weeks. Moreover, one group with live *L. acidophilus* was treated at 12 weeks with LPS. The amount of *L. acidophilus* was significantly reduced in the feces of with HFD *ApoE*^−/−^ mice compared the mice with NCD (Fig. 7A). The live *L. acidophilus* (5 × 10^9^ CFU) can restore the diminished level by the HFD by oral gavage daily. However, lipopolysaccharide reversed the amount of *L. acidophilus* (Fig. 7A).

**Fig 8:**
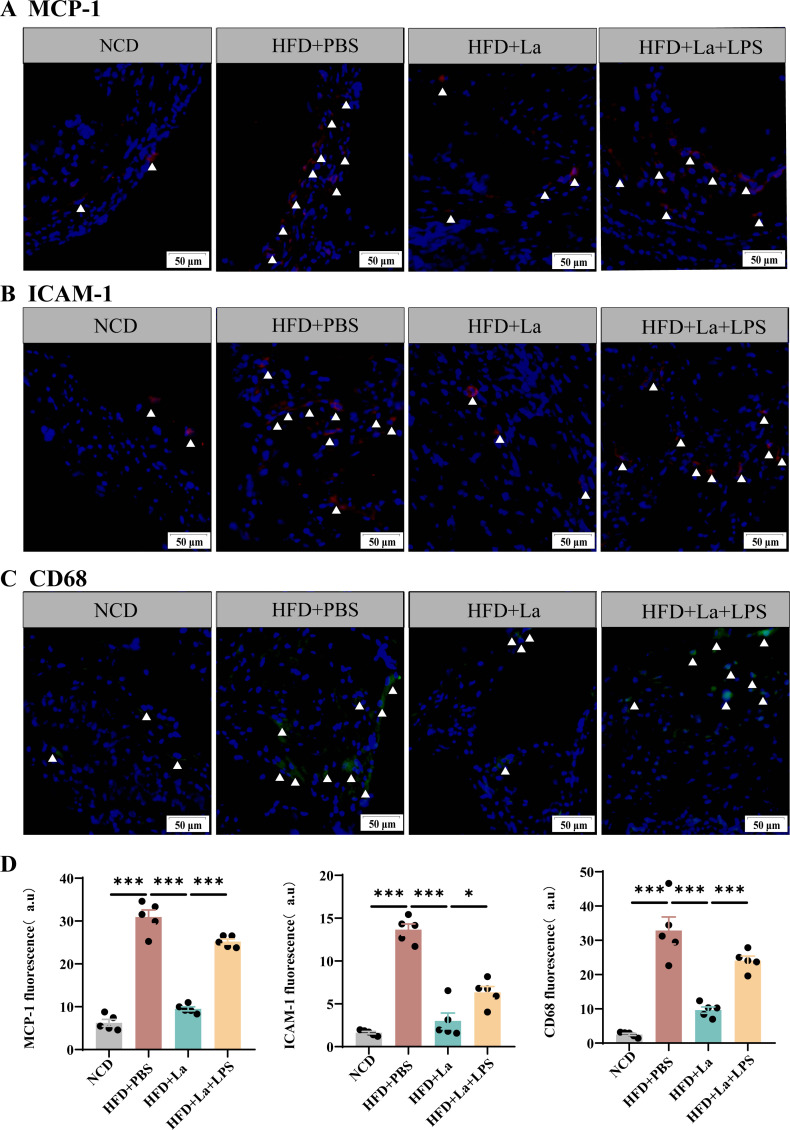
*L. acidophilus* attenuated systemic inflammation LPS reversed the process. (**A–C**) The protein expression of MCP-1, ICAM-1, and CD68. (**D**) Quantitative analysis of images from panels A–C was performed. Representative images of each group are shown. Data are shown as the mean ± SEM (*P < 0.05, **P < 0.01, ***P < 0.001) by one-way analysis of variance and Tukey’s post hoc analysis. LPS, lipopolysaccharide.

The Oil Red O staining of aortas and aortic root regions and the hematoxylin-eosin staining of aortic root regions were evaluated for the formation of atherosclerotic lesions by the high-fat diet (Fig. 7B and D). The *ApoE*^−/−^ mice with HFD had a significantly increased the lesion area and necrotic core area compared with the mice with the normal chow diet (Fig. 7C and E). The lesion areas and necrotic core area were significantly reduced with *L. acidophilus* treatment in aortas and aortic root regions (Fig. 7F and G). However, the presence of lipopolysaccharide counteracted the beneficial impact of *L. acidophilus* intervention on the prevention of atherosclerosis. Lipopolysaccharide increased the lesion area and necrotic core area in *ApoE^-/-^* mice compared with the *L. acidophilus*-treated mice (Fig. 7B through F). *L. acidophilus* inhibits the expression of pro-inflammatory factors and promotes the expression of inhibitory factors (Fig. 7H). Moreover, *L. acidophilus* exhibited a reduction in the infiltration of macrophages (CD68), as well as the inflammatory molecules MCP-1 and ICAM-1, within the atherosclerotic lesions in *ApoE*^−/−^ mice. However, the presence of lipopolysaccharide reversed this effect (Fig. 8A through D).

### *L. acidophilus* improves intestinal permeability

**Fig 9:**
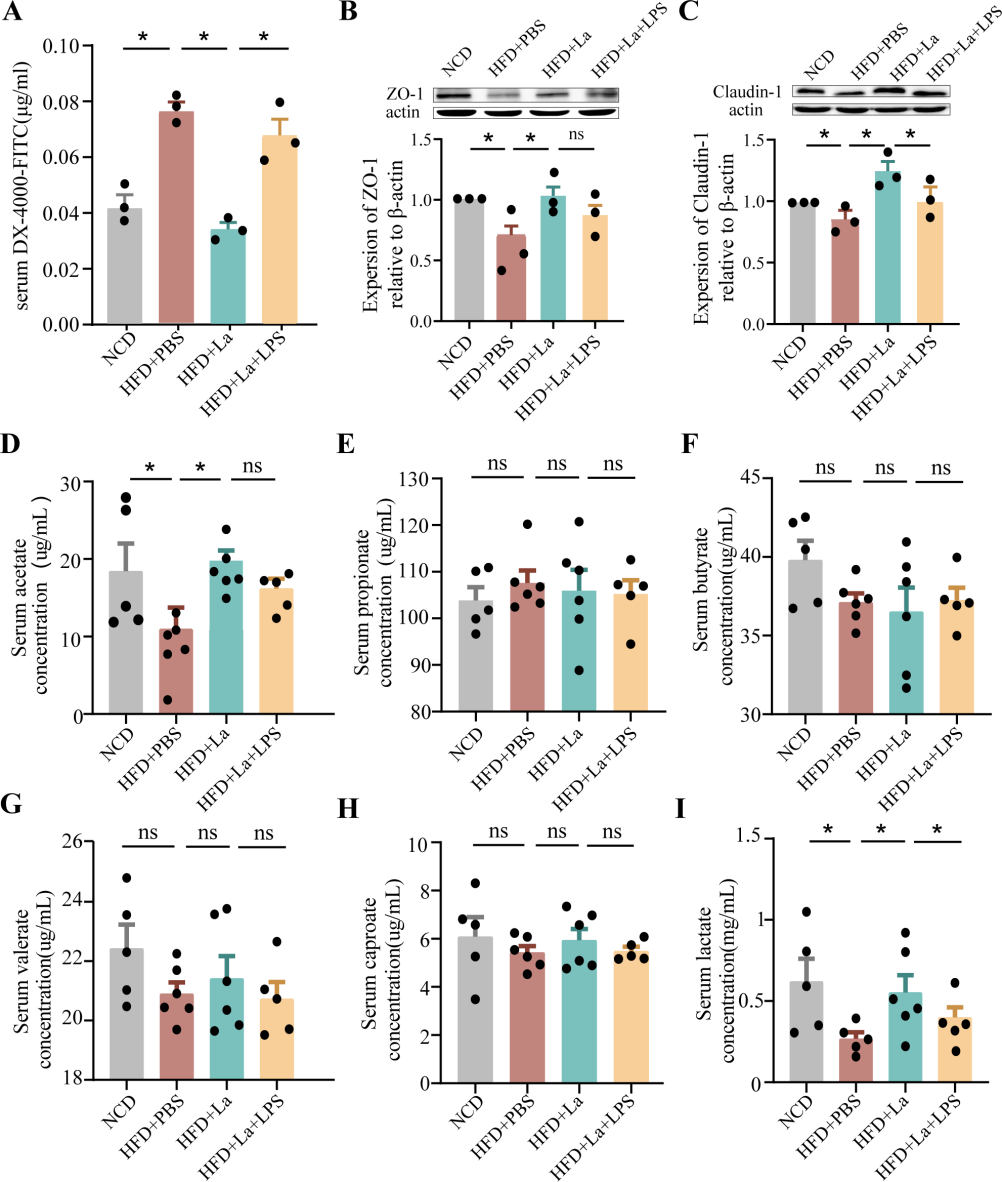
*L. acidophilus* improves intestinal permeability and enhances expression of intestinal tight junction proteins. (**A**) Quantification of serum FITC-dextran. *n* = 6. (**B and C**) ZO-1 and claudin-1 expressions were determined with Western blot and normalized to actin. *n* = 3. (**D–I**) The relative level of SCFAs in serum content was measured through gas chromatography–mass spectrometry. *n* = 5. Data are shown as the mean ± SEM (*P < 0.05, **P < 0.01, ***P < 0.001) by one-way analysis of variance and Tukey’s post hoc analysis.

To determine whether *L. acidophilus* can improve intestinal permeability, the concentration of fluorescent-labeled dextran (DX-4000-FITC) in plasma was detected in *ApoE*^−/−^ mice. The values were found to be significantly elevated in HFD-fed *ApoE*^−/−^ mice as compared to mice fed with NCD. *L. acidophilus* decreased the gut permeability, whereas lipopolysaccharide blocked the reduction (Fig. 9A), which suggests that *L. acidophilus* preserves the gut barrier by blocking the penetration of lipopolysaccharide. The expression of the epithelial tight junction protein ZO-1 exhibited a significant decrease in *ApoE*^−/−^ mice fed HFD compared to *ApoE*^−/−^mice on NCD. *L. acidophilus* increased the expression of the epithelial tight junction protein ZO-1 and claudin-1, but lipopolysaccharide blocked it (Fig. 9B and C). The short-chain fatty acids (SCFAs) were measured in the serum by gas chromatography–mass spectrometry. The levels of acetate and lactate showed a substantial increase in *ApoE*^−/−^ mice fed HFD supplemented with *L. acidophilus*, in comparison to those fed a Western diet supplemented with PBS (Fig. 9D through I).

## DISCUSSION

Atherosclerosis is generally caused by an imbalance between lipid metabolism and cholesterol-rich macrophage accumulation in arterial walls (21). CAD is caused by the blockage of the coronary arteries that supply blood to the heart due to atherosclerosis, which can lead to symptoms, such as angina and myocardial infarction. Although atherosclerosis is the primary driver of CAD, evaluation and treatment approaches have historically relied on indirect markers of atherosclerosis (22). In the present study, 202 DEGs were identified between 117 CAD and 79 healthy control samples. Considering the high expression of *eIF6* in patients with CAD, we aimed to determine whether *eIF6* is highly expressed in atherosclerotic models and whether it ultimately contributes to CAD.

Gut microbiota has been shown to be significantly associated with cardiovascular diseases (23). Modulating the gut microbiota in low-density lipoprotein receptor null mice leads to a decrease in the progression of atherosclerosis (24). The prominent bacterium *Akkermansia muciniphila* has been shown to serve as a protective shield against atherosclerosis in *ApoE*^−/−^ mice by effectively reducing inflammation caused by metabolic endotoxemia (25). In the present study, *eIF6* deficiency alters the composition of gut microbiota in mice. The abundance of *Lactobacillus* and *Bifidobacterium* was significantly increased. However, the abundance of *Akkermansia* was decreased. *eIF6* deficiency may have led to the increase of some beneficial bacteria and the decrease of some beneficial bacteria. We can draw the conclusion that *eIF6* deficiency changes the microenvironment of intestinal flora, which in turn increases the abundance of some probiotics.

The gut microbiota plays a crucial role in host metabolism and immune regulation, influencing lipid metabolism and inflammatory processes associated with atherosclerosis (26, 27). However, the role of *eIF6* in the regulation of the gut microbiota in atherosclerosis has not yet been fully confirmed. In this study, fecal microbiota analysis of *eIF6*^+/−^ and ApoE^−/−^/*eIF6*^+/−^ mice revealed that *eIF6* increased the abundance of several beneficial bacteria, thereby optimizing and altering the gut microbiota composition. To explore the role of *eIF6* in regulating gut microbiota on atherosclerosis, we constructed a double knockout mouse model, *ApoE*^−/−^/*eIF6^+/^*^−^ mice. The abundance of *Lactobacillus*, *Ileibacterium* and *Bifidobacterium* increase in the *ApoE*^−/−^/*eIF6^+/^*^−^ mice with HFD compared with the *ApoE*^−/−^ mice. It is well known that prebiotics have been usually used to support the body’s overall health, and it can enhance the diversity and abundance of gut microbiota, thereby preventing certain diseases (28, 29). High-fat diet leads to a decrease in gut *Lactobacillus* and aggravates atherosclerosis. With oral supplementation of *L. acidophilus*, the development of atherosclerosis is reduced. Therefore, focusing on targeting this single genus/species in the gut microbiota shows great promise as an effective intervention for preventing the development of atherosclerosis.

Atherosclerosis is a chronic inflammatory disease, with increased inflammation being a key causal factor. Targeting inflammatory could provide a promising and innovative approach for the prevention and treatment of atherosclerosis (30). Macrophages engulf oxidized low-density lipoprotein and clear it from the vascular endothelium. When macrophages become “full” due to phagocytosis of excess lipids, resulting in dysregulation of lipid metabolism, eventually forming foam cells, which lead to inflammation (3). The current clinical drugs for the treatment of atherosclerosis include aspirin, urinary/streptokinase, recombinant tissue plasminogen activator, statins, angiotensin transferase inhibitors (31, 32). The risks of developing fatal cardiovascular complications in cardiovascular patients remain elevated due to the persisting unresolved inflammation. The value of Firmicutes/Bacteroidetes increases in *ApoE^−/−^*mice with Western diet, indicating the inflammation increases (25). *eIF6* deficiency can inhibit the level of inflammation factors (14). In this study, the values of Firmicutes/Bacteroidetes decrease in the *ApoE*^−/−^/*eIF6^+^*^/−^ mice with HFD compared with *ApoE*^−/−^mice. The administration of *L. acidophilus* in *ApoE^−/−^* mice led to a notable reduction in the expression of CD68, MCP-1 and ICAM-1. The aortic infiltration of inflammatory cells and atherosclerotic lesion also diminished. *L. acidophilus* exerts anti-inflammatory effects in atherosclerosis.

Alterations in gut microbiota led to microbiota-associated metabolic dysfunction, and these metabolites play a vital role in upholding the overall well-being of the cardiovascular system. The metabolites with high attention include TMAO, SCFAs, bile acids, and lipopolysaccharide (33). *eIF6* deficiency in *ApoE*^−/−^ mice altered the metabolites, and we further analyzed the correlation between gut microbiota, metabolites, metabolic parameters, and atherosclerotic markers. KEGG enrichment revealed *eIF6* deficiency in *ApoE^-/-^* mice affected retrograde endocannabinoid signaling, ether lipid metabolism, alpha-linoleic acid metabolism, and linoleic acid metabolism. Ether lipid metabolism has a beneficial effect on platelet function and thrombosis (34). Linoleic acid plays a significant role in mitigating the risk of coronary heart disease and associated mortality by lowering serum cholesterol (35). Therefore, *eIF6* deficiency reduced lipid absorption and protected from high-fat diet-induced atherosclerosis.

LPS is a crucial metabolite produced by gut microbiota. It not only triggers the release of inflammatory cytokines in perivascular adipose tissue but also impedes cholesterol efflux from macrophages, thereby promoting the formation of foam cells (36). *L. acidophilus* inhibits body weight, fat mass, inflammation and insulin resistance in mice fed with HFD (37); gavaging with *L. acidophilus* reduced pro-inflammatory cytokines. *L. acidophilus* reduced pro-inflammatory cytokines, promoted the increase of goblet cells and the secretion of antimicrobial peptides, regulated the ratio of Firmicutes/Bacteroidetes, and increased the level of acetate (37).

In this study, *L. acidophilus* alleviated high-fat diet-induced atherosclerosis, but lipopolysaccharide reversed the process, indicating that *L. acidophilus* can limit the lipopolysaccharide level in the bloodstream, further showing an antiatherogenic effect. SCFAs are end products of bacterial metabolism in the intestine that play a vital role in promoting cardiovascular health in the host (38). Administration of *L. acidophilus* increased the content of acetate and lactate in the study. Therefore, the augmented SCFA production mediates suppression of inflammation and benefits atherosclerosis.

Intestinal permeability relies on a healthy epithelial lining. When the permeability of the intestines increases, antigens, toxins, and pathogens can infiltrate the mucosal tissue, leading to inflammation (39).The tight junction protein determines the permeability of the intestine, including occludin, claudins, and Zos (40). The levels of the two tight junction proteins, occludin-1 and ZO-1, showed an elevation in the ileum of *ApoE^−/−^* mice following the administration of *L. acidophilus*. Moreover, the integrity of the intestinal mucosal barrier, which was compromised by HFD and LPS, was recovered. The FITC-dextran test has already obtained similar results.

### Conclusion

In conclusion, our study demonstrated that *eIF6* deficiency regulates the gut microbiota and a wide range of metabolites in atherosclerosis *ApoE^−/−^* mice. *L. acidophilus* decreased in the gut of atherosclerosis *ApoE^−/−^* mice, but administration of *L. acidophilus* could reverse the gut barrier dysfunction and vascular inflammation. Our findings indicate that targeting individual species is a beneficial treatment strategy to protect against inflammation and atherosclerosis.

## Data Availability

The original contributions presented in the study are included in the article. Further inquiries can be directed to the corresponding authors. The original data can be found in https://figshare.com/articles/dataset/eIF6_/24311707.

## SUPPLEMENTAL MATERIAL

Supplemental Figures msystems.00595-24-s0001.pdf Figures S1 and S2.

Legend msystems.00595-24-s0002.docx Legend for original western blots.

Blots msystems.00595-24-s0003.pdf Original western blots.

ASM does not own the copyrights to Supplemental Material that may be linked to, or accessed through, an article. The authors have granted ASM a non-exclusive, world-wide license to publish the Supplemental Material files. Please contact the corresponding author directly for reuse.
