# Supplementary material for: eIF6 deficiency regulates gut microbiota, decreases systemic inflammation, and alleviates atherosclerosis
Source: mSystems. 2024 Sep 3;9(10):e00595-24. doi: 10.1128/msystems.00595-24 (PMC11494895; doi:10.1128/msystems.00595-24)
Supplement: Supplemental Figures — Figures S1 and S2. [file msystems.00595-24-s0001.pdf]

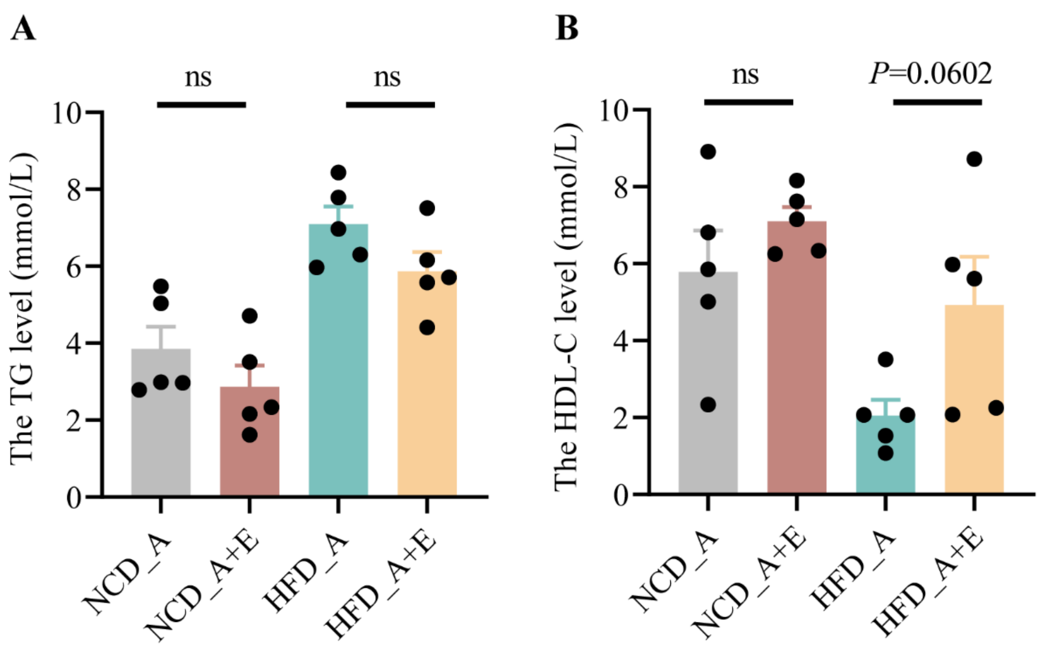

Figure S1 *eIF6* deficiency reduced serum lipid metabolism. (A) Serum total cholesterol (TC), (B) Serum high-density lipoprotein cholesterol (HDL-C). Datas are shown as the mean ± SEM, **P*<0.05, ***P*<0.01, ****P*<0.01 by one way ANOVA and Tukey post hoc. (NCD_A, NCD_*ApoE*$^{-/-}$, NCD_A+E, NCD_*ApoE*$^{-/-}$/*eIF6*$^{+/-}$, HFD_A, HFD_*ApoE*$^{-/-}$, HFD_A+E, HFD_*ApoE*$^{-/-}$/*eIF6*$^{+/-}$).

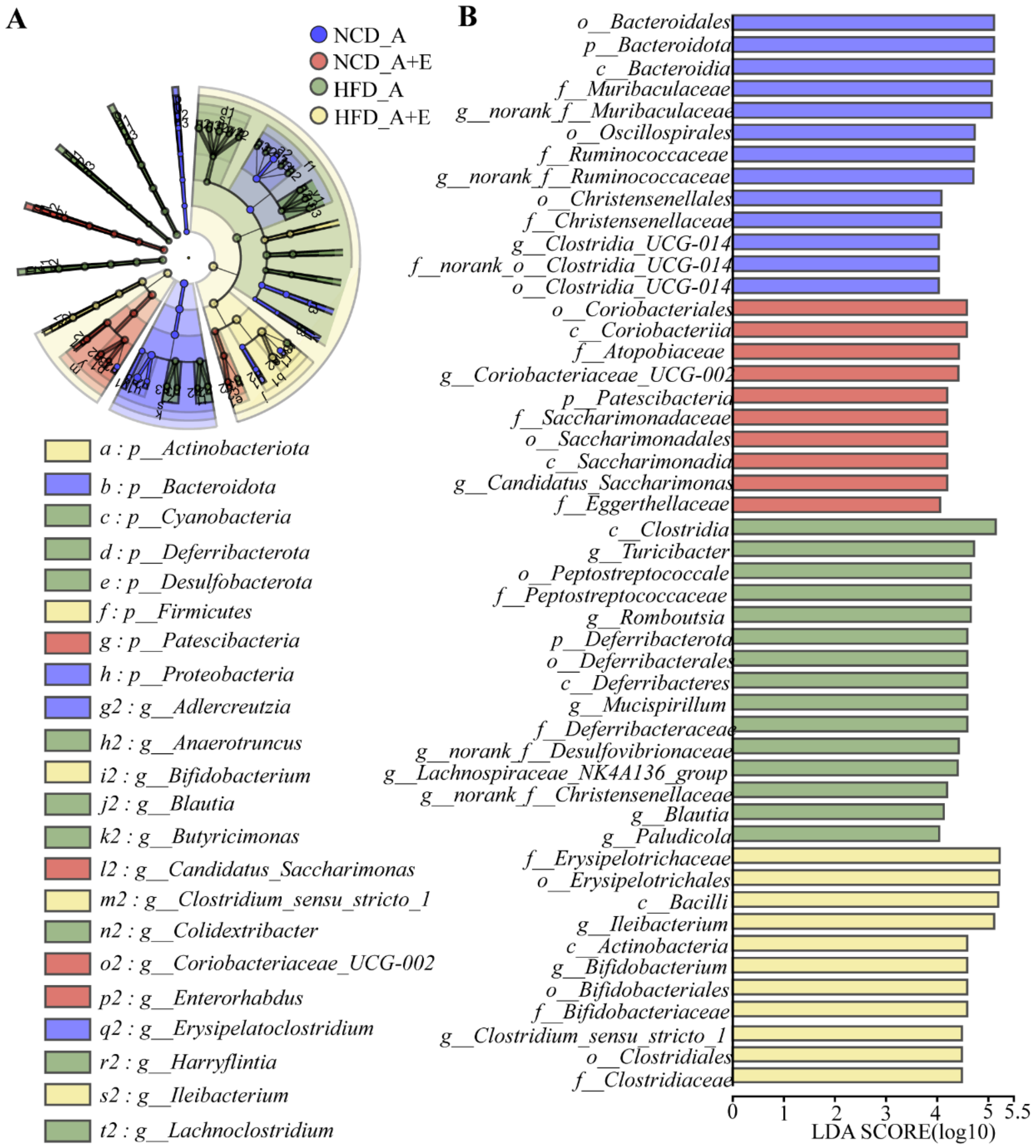

Figure S2 Taxonomic cladogram obtained using LEfSe analysis of the 16S rRNA sequences. (A) Cladogram generated from linear discriminant analysis effect size (LEfSe) showing the most diferentially enriched bacterial taxa in the fecal microbiota of NCD_A (blue), NCD_A+E (red), HFD _A (green), or HFD _A+E (yellow) mice (LDA value= 4; *P* value< 0.05). (B) Computed LDA scores of the relative abundance difference among the NCD_A (blue), NCD_A+E (red), HFD _A (green), or HFD _A+E (yellow) mice.
