# Supplementary material for: eIF6 deficiency regulates gut microbiota, decreases systemic inflammation, and alleviates atherosclerosis
Source: mSystems. 2024 Sep 3;9(10):e00595-24. doi: 10.1128/msystems.00595-24 (PMC11494895; doi:10.1128/msystems.00595-24)
Supplement: Legend — for original western blots. [file msystems.00595-24-s0002.docx]

Claudin-1

We detected the Claudin-1 expressions by Western blot in the intestinal endothelium of mice (NCD, HFD+PBS, HFD+La, HFD+La+PBS), Claudin-1 expressions normalized to actin. Each band shows the results of the test after mixing three different mouse samples. We repeated it three times.

ZO-1

We detected the ZO-1 expressions by Western blot in the intestinal endothelium of mice (NCD, HFD+PBS, HFD+La, HFD+La+PBS), ZO-1 expressions normalized to actin. Each band shows the results of the test after mixing three different mouse samples. We repeated it three times.
