# Supplementary figures and images for: eIF6 deficiency regulates gut microbiota, decreases systemic inflammation, and alleviates atherosclerosis
Source: mSystems. 2024 Sep 3;9(10):e00595-24. doi: 10.1128/msystems.00595-24 (PMC11494895; doi:10.1128/msystems.00595-24)

Claudin-1

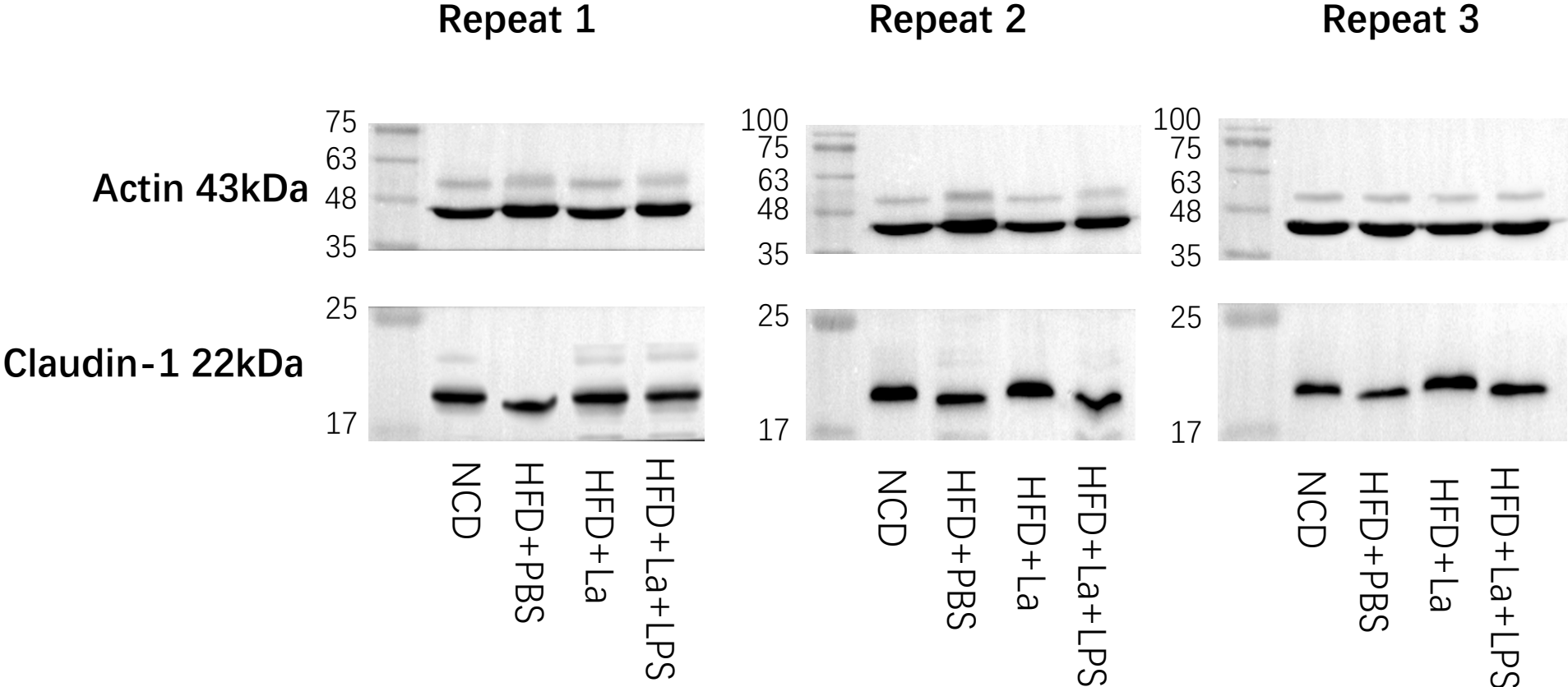

Zo-1

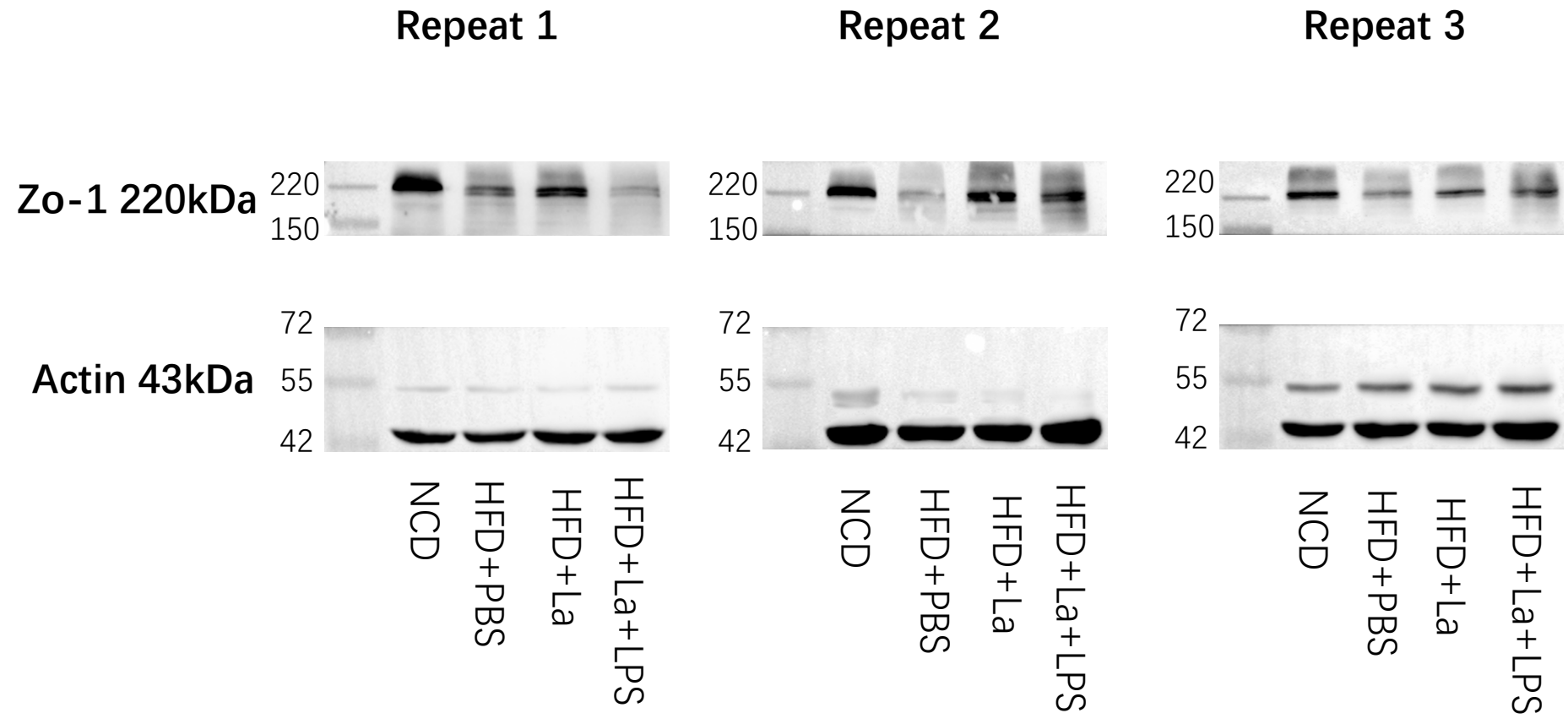

Supplement: Blots — Original western blots. [file msystems.00595-24-s0003.pdf]
